# Control of Bacterial Diseases of Banana Using CRISPR/Cas-Based Gene Editing

**DOI:** 10.3390/ijms23073619

**Published:** 2022-03-25

**Authors:** Leena Tripathi, Valentine O. Ntui, Jaindra N. Tripathi

**Affiliations:** International Institute of Tropical Agriculture (IITA), Nairobi P.O. Box 30709-00100, Kenya; v.ntui@cgiar.org (V.O.N.); j.tripathi@cgiar.org (J.N.T.)

**Keywords:** banana, bacterial diseases, banana Xanthomonas wilt, gene editing, disease resistance

## Abstract

Banana is an important staple food crop and a source of income for smallholder farmers in about 150 tropical and sub-tropical countries. Several bacterial diseases, such as banana Xanthomonas wilt (BXW), blood, and moko disease, cause substantial impacts on banana production. There is a vast yield gap in the production of bananas in regions where bacterial pathogens and several other pathogens and pests are present together in the same field. BXW disease caused by *Xanthomonas campestris* pv. *musacearum* is reported to be the most destructive banana disease in East Africa. The disease affects all the banana varieties grown in the region. Only the wild-type diploid banana, *Musa balbisiana*, is resistant to BXW disease. Developing disease-resistant varieties of bananas is one of the most effective strategies to manage diseases. Recent advances in CRISPR/Cas-based gene editing techniques can accelerate banana improvement. Some progress has been made to create resistance against bacterial pathogens using CRISPR/Cas9-mediated gene editing by knocking out the disease-causing susceptibility (S) genes or activating the expression of the plant defense genes. A synopsis of recent advancements and perspectives on the application of gene editing for the control of bacterial wilt diseases are presented in this article.

## 1. Introduction

Banana and plantain (*Musa* spp.), hereafter collectively referred to as banana, originated from Southeast Asia and the Pacific and are now cultivated in about 150 nations in the tropics and subtropics on 12 million hectares of land [1]. It is a key food security and cash crop with an annual production of over 163 million tons feeding millions of people [1]. Africa produces one-third of the global banana, with East Africa the leading producer accounting for up to 40% of the total African production [1]. The Great Lakes region of Africa (GLA), containing Burundi, Kenya, the eastern part of the Democratic Republic of Congo (DRC), Rwanda, Tanzania, and Uganda, is the largest banana-growing and -consuming region with a consumption rate of 220–460 kg per person annually, which is six times Africa’s average and 15 times the world’s average [2]. Banana provides 30–60% of these countries’ daily per capita calorie intake. According to data from the Food and Agriculture Organization (FAO), the GLA region accounts for approximately 60% of the total banana cultivation area in Africa [2]. Banana is rich in vitamins, minerals, and carbohydrates and is considered one of the primary energy sources for millions of people in East Africa.

Banana is grown mainly by smallholder farmers for domestic consumption and local or regional markets, and less than 15% enter the international markets. Smallholder farmers grow different banana varieties, such as dessert, cooking, roasting, and brewing types. However, for wholesale production, farmers mainly grow the Cavendish (AAA genome) as dessert types of banana. Other dessert types such as Sukali Ndiizi (AAB genome, commonly known as apple banana), Silk (AAB genome), Pome (AAB genome), and Mysore (AAB genome), are also grown at a low level in various regions as dessert banana. Aside from that, cooking types like the East African Highland Banana (EAHB, AAA genome) and Bluggoe (ABB genome), roasting types like plantain (AAB genome), and brewing types like Pisang Awak (ABB genome) are primarily cultivated in Africa.

Several diseases and pests wreak havoc on banana production, especially in areas where multiple pests and pathogens coexist [3]. Several bacterial diseases, such as banana Xanthomonas wilt (BXW), blood, and moko disease, cause significant yield losses [4]. It can lead to a significant yield gap in banana production, especially in locations where bacterial infections, as well as a variety of other pathogens and pests, are present. For example, BXW, in combination with fungal and viral pathogens, nematodes, and weevils, is devastating banana production in the GLA region. The yield gap in banana production needs to be closed urgently, which can improve food security, particularly in Africa, where it feeds more people per unit area of production when compared to other staple crops [5].

**Figure 1:**
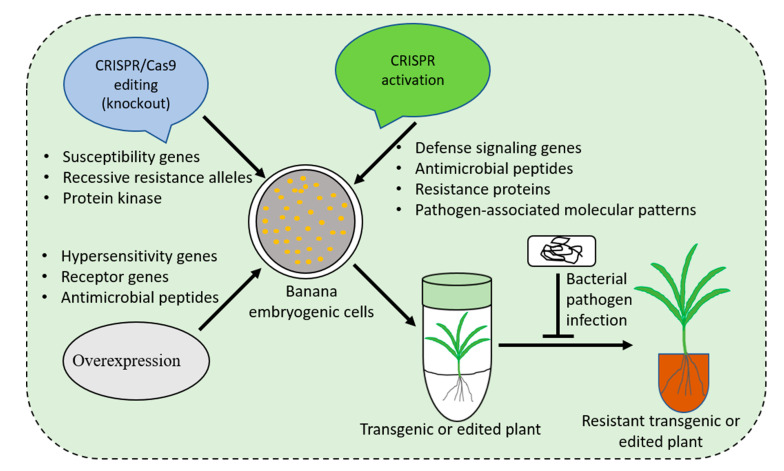
Schematic illustration of strategies for developing bacterial disease(s) resistant banana varieties.

Developing disease-resistant banana varieties using traditional breeding is challenging because of the low genetic variability available in *Musa* germplasm, lengthy production cycle, polyploidy, and sterility of most of the cultivars [3]. Modern breeding tools, including transgenics and gene editing, present the potential to complement conventional breeding for developing disease resistance in farmer-preferred banana varieties (Figure 1). Intensive efforts have been made to generate banana cultivars with enhanced resistance to bacterial wilt disease through genetic manipulation (GM). The commercialization of GM crops, however, is hampered by the lengthy regulatory procedures. Gene editing, a powerful emerging tool, could be applied for developing durable resistance to various diseases. Some progress has been made in banana improvement to create resistance against bacterial pathogens using gene editing. Here, we describe an overview of current advances and future perspectives for using gene editing to generate bacterial disease-resistant banana.

## 2. Bacterial Diseases of Banana

Bacterial diseases of banana are among the most damaging, resulting in considerable yield reductions. Several bacterial pathogens are reported to cause substantial impacts on banana production. The major bacterial diseases include banana Xanthomonas wilt (BXW), caused by *Xanthomonas campestris* pv. *musacearum* (Xcm), moko and bugtok disease, caused by *Ralstonia solanacearum*, and blood disease, caused by *Ralstonia syzygii* subsp. *celebesensis* [6]. 

The fungal diseases such as black Sigatoka (caused by *Pseudocercospora fijiensis*) and fusarium wilt (caused by *Fusarium oxysporum* f. sp. *cubense*) are considered as the most important diseases affecting banana production, and their management has gained increasing attention compared to other diseases [6]. However, the management practices for controlling bacterial diseases are not well known even though the bacterial diseases continue to cause significant losses in banana production globally. Only substantial efforts are put in place to control banana Xanthomonas wilt disease. More efforts are needed to prevent bacterial diseases by integrating molecular approaches with conventional breeding to develop disease-resistant varieties, which is a cost-effective, less labor-intensive, and environmentally friendly option.

## 3. Banana Xanthomonas Wilt (BXW)

BXW is a major bacterial disease that reduces yield and raises crop management costs in East Africa. The BXW disease affects the production of all different types of bananas grown in East Africa, and its effects are severe and swift, wiping out whole plantations in many of the affected locations. The disease can cause up to 100% yield losses, mainly in brewing type banana, severely affecting food security and livelihoods for banana farmers [7]. BXW was first reported on Ensete (*Ensete ventricosum*) in Ethiopia in the 1930s. Since 2001, the disease has spread to commercially cultivated banana in Burundi, Kenya, Rwanda, the Democratic Republic of Congo, Tanzania, and Uganda [8]. BXW disease has made a huge impact on the food security and income of small-scale farmers, who rely on banana for a living. The estimated economic losses due to BXW disease are at US$ 2 to 8 billion over a decade [8]. The bacterial pathogen is transmitted by insects that perch on the male inflorescence, infected planting material, contaminated tools, and infected banana tissues such as leaves and pseudostem sheaths. Different approaches have been explored as an intervention towards controlling the deadly disease. Phytosanitary methods such as using clean pathogen-free planting material, decapitating male buds, using clean, sterile gardening tools, cutting and burying infected plants, and restricting the transportation of banana materials from BXW-affected areas are some of the ways employed to manage BXW disease. However, because such procedures are labor-intensive, they have been inconsistently adopted. 

The pathogen’s resistance has recently been identified in *Musa balbisiana* and *Musa acuminata* subsp. *zebrina* [4,9]. The banana varieties with morphological traits such as persistent- bracts or the absence of the male buds help them avoid insect-mediated infection [10]. Transgenic approaches and gene editing were recently used to develop resistance to the pathogen on banana cultivar Sukali Ndiizi [11]. 

## 4. Moko and Bugtok Disease

Moko disease was first reported in the 1890s in banana in Trinidad [12]. Several banana varieties were infected with this disease. Still, the disease was found to be severe in the cooking type banana Bluggoe (ABB) (also known as Moko), from which the disease’s common name was derived. Moko disease is present mainly in Latin America, including Belize, Brazil, Colombia, Costa Rica, Ecuador, El Salvador, Grenada, Grenadines, Guatemala, Guyana, Honduras, Jamaica, Mexico, Nicaragua, Panama, Peru, St. Vincent, Suriname, Trinidad and Tobago, and Venezuela. Moko disease has also been detected in the Southern Mindanao part of the Philippines. This disease in the Philippines might have been introduced through the infected planting materials of the ‘Valery’ variety (AAA Cavendish subgroup) [13]. 

Moko disease is considered a severe banana disease, and its control is expensive. In some cases, the yield loss can reach up to 100%. For example, in Colombia, losses of up to 100% were recorded in some plantations [14]. The disease symptoms start with young leaves wilting, which later die and collapse. The petiole weakens, resulting in drooping green leaves and a decline in tree vigor. Older leaves are also impacted as the disease progresses. As the pulp is destroyed by dry rot, infected fruits develop distorted and shrivel up, resulting in dark brown discoloration of the fruit flesh. The disease management strategy includes using pathogen-free clean planting material, avoiding infection through regular decontamination of field instruments, and removal of diseased banana-mat. Since the disease is also soil-borne, a 6-month delay is required before replanting [10].

The disease caused by *R*. *solanacearum* is called Bugtok when the plants show wilt symptoms. The pathogen is transmitted mainly by insects and affects the cooking type (ABB) banana in the Philippines [15]. In the early 1990s, it was a dominant disease of the Saba (BBB) variety. 

## 5. Blood Disease

Banana blood disease was first reported on dessert banana in the early 1900s in Salayar Island near Sulawesi [16]. The disease was confined to Selayer and Southwestern Sulawesi until 1987, when the disease was reported in West Java and quickly spread to other islands in Indonesia, including the Indonesian archipelago, Kalimantan, Sumatra, Sumbawa, Bali, Maluku, West Papua, and recently to the Malaysia peninsular [10,17]. Severe yield losses due to banana blood disease have been noted in several areas. For example, in South Sulawesi, 70–80% of plantations were lost [18], and in West Java, 27–36% plantation loss was recorded [19]. In Lampung Province (Sumatra), more than 20,000 tons of banana, worth US$ 1 million were lost [20]. Currently, there are no sources of resistance, and management of the disease includes using pathogen-free clean planting material and the regular decontamination of field instruments to avoid infections, which are labor-intensive and costly.

## 6. Recent Advances in Gene Editing of Banana

Gene editing, a powerful emerging tool, can develop durable resistance to diseases. Recent advances in gene-editing technologies using site-directed nucleases (SDNs), such as meganucleases, zinc-finger nucleases, transcription activator-like effector nucleases (TALENs), and the clustered regularly interspaced short palindromic repeats/CRISPR-associated protein (CRISPR/Cas). The CRISPR/Cas, derived from the adaptive immune system of *Streptococcus pyogenes*, have promoted the manipulation of genes in several crop species [21]. CRISPR/Cas is the most potent and desired tool for crop gene editing as it is easy to design reagent, has high efficacy, and can edit numerous genes simultaneously [3]. 

CRISPR/Cas9 comprises of the synthetic guide RNA (sgRNA) and the Cas9 nuclease. Cas9 recognizes target DNA by matching the 5′ leading sequence of sgRNA with the 5′ leading sequence of DNA. It detects the protospacer adjacent motif (PAM), a three-nucleotide sequence, mostly NGG or NAG (where N is any nucleotide), that serves as a recognition segment for Cas9 to start checking the upstream sequence against the gRNA. It is generally found 3–4 nucleotides downstream from the cut site. The sgRNA involves a scaffold and a spacer sequence of about 20 nucleotides for targeting the genomic sequence. It directs the Cas9 to create precise and targeted double-stranded breaks (DSBs). Then, the DSBs at the target site is repaired either by the non-homologous end-joining (NHEJ) or homology-directed repair (HDR) if a donor template is available, resulting in small deletions or insertions, substitutions of nucleotides, or gene replacement.

Apart from Cas9, other Cas proteins have been developed and used to edit plants. Cas12a (Cpf1) is a class 2, type V-CRISPR, which harbours the RuvC domain only. It possesses crRNA biogenesis RNase and single-strand DNase activity and recognizes T-rich PAM, TTN/TTTN/TTTV (N = A/T/C/G; V = A/C/G). Cas12a could be used for gene editing for multiplexing by a single sequence array on the selected sgRNA. Cas13a is a class 2 type VI-A ribonuclease that can target and cleave single-stranded RNA (ssRNA) molecules of the phage gene [22]. It could be used for detecting RNA viruses as it is more accurate than PCR in detecting viruses. Cas13a does not require a PAM segment.

Based on the repair mechanism, three types of gene editing systems, SDN1, SDN2, and SDN3, have been identified [21,23]. SDN1 is a very efficient, error-prone repair of a targeted DSB that is based on NHEJ. The DSB repair causes random changes in the host genome, which can result in gene silence, knockout, or altered function. SDN2 is less efficient and high fidelity and consists of a repair template with sequence identity to the target site added to the CRISPR/Cas reagent. The DSB in the SDN2 is subsequently repaired via HDR, which results in nucleotide substitution or targeted indels. SDN3 has high fidelity but less efficient enzymatic activity. The DSB in SDN3 is repaired via HDR using the donor template, resulting in the insertion of the entire gene or genetic element(s) at the target site based on donor sequence. Another type of editing system is base editing (BE), used for target editing of a single base pair. It requires the fusion of DNA deaminase to dCas9 to generate a base editor that allows single-nucleotide base substitution resolution without a DNA donor template. The DNA deaminases act as effectors, allowing C:G-to-T:A or A:T-to-G:C substitution depending on the types of DNA deaminases used, whereas the RNA-guided CRISPR system serves as a genomic locator of the targeted region. 

Recently, prime editing was developed as another SDN editing tool. Prime editing uses the exact mechanism as classical CRISPR/Cas systems, mediating DNA base pair substitutions, small insertions, small deletions (indels) [24]. However, primer editing does not induce DSB and does not require a donor template; it resolves frameshifts induced by indels and minimizes off-target effects. To edit a genome, prime editing requires a longer-than-usual sgRNA, known as pegRNA, and a fusion protein comprising of Cas9 H840A nickase fused to an engineered RT enzyme. Although prime editing allows for precise and targeted modifications in DNA and has the potential to complement the existing CRISPR editing systems, its cellular determinants remain poorly understood. Nevertheless, prime editing is an exciting tool that could be exploited for banana gene editing. Since base editing and prime editing do not require a DNA donor template, they might be considered as SDN1 and SDN2 types of gene editing, respectively, which can be treated like non-transgenic products and do not require biosafety regulations similar to transgenic products [3].

The availability of a robust genetic transformation protocol and the whole-genome sequence makes the banana an excellent candidate for gene editing. Banana gene editing was first reported in the cultivar “Rasthali” (AAB genome) by targeting the *phytoene desaturase* (PDS) as a marker gene [25]. The authors used a single sgRNA to generate mutations in the PDS gene, producing an albino phenotype. However, the mutation efficiency was only 59%. Further, Naim et al. [26] reported editing of the PDS gene in “Cavendish Williams” (AAA genome) with a 100% editing efficiency using polycistronic tRNA. Similarly, Ntui et al. [27] reported 100% mutation efficiency in banana cultivar “Sukali Ndiizi” (AAB genome) and plantain cultivar “Gonja Manjaya” (AAB genome) using multiple sgRNAs targeting the PDS gene. The PDS is one of the main enzymes in the carotenoid biosynthesis pathway commonly used as a marker to optimize gene editing. It encodes for a crucial enzyme that converts phytoene to carotenoid precursors phytofluene and ζ-carotene in the pathway. When its function is disrupted, phenotypes which include albino, variegated or pale-green, depending on the mutation pattern of the transformed plant, are produced and are detected with the naked eyes. However, PDS knockout negatively impacts plant development. Therefore, as an alternative, Zorrilla-Fontanesi et al. [28] edited RP43/CHAOS39, a gene that encodes the chloroplast signal recognition particle (cpSRP) machinery, as a visual marker to optimize gene editing procedures in banana. The CHAOS39-edited banana plants were pale-green and grew normally. Nevertheless, researchers must exercise caution when employing cpSRP43/CHAOS39 as a visual marker because the pale green phenotype can be obtained by other factors such as insufficient light and nutrient deficiency, for example, iron and magnesium involved in chlorophyll molecule structure and photosynthesis. 

One of the most important applications of gene editing is to develop disease-resistant plants to increase their economic values, enhancing nutrition and food security. Some progress has been demonstrated for creating resistance to banana streak virus (BSV) and BXW disease. The endogenous banana streak virus (eBSV) integrated into the B genome of plantain (AAB) was inactivated using CRISPR/Cas9-based editing to overcome a key barrier in breeding and the distribution of hybrids [29]. BSV is a dsDNA badnavirus, whose genome gets integrated into the B genome of plantain. The gene-edited plantain “Gonja Manjaya” had targeted mutations in the eBSV sequences integrated into the host genome. Phenotyping of the edited events varified the inactivation of eBSV for its ability to generate functional infectious viral particles. Recently, it was demonstrated that the editing of *MusaDMR6* in banana using CRISPR/Cas9 mediated gene editing resulted in enhanced resistance to BXW disease [11]. 

In addition to developing disease resistance, gene editing has also been used to increase banana fruit quality, shelf-life, and alter the plant architecture. For example, the CRISPR/Cas9 was used to generate β-carotene-enriched Cavendish banana cultivar “Grand Naine” by editing the *lycopene epsilon-cyclase* (*LCY*ε) gene, which converts lycopene to delta-carotene and neurosporene to alpha-zeacarotene and is required for lutein biosynthesis [30]. CRISPR/Cas9 technology was applied to generate semi-dwarf banana cultivar “Gros Michel” by manipulating the *M. acuminata gibberellin* 20ox2 (MaGA20ox2) gene, disrupting the gibberellin (GA) pathway [31]. GA is an important gene determining plant height, and mutations in its biosynthesis genes usually produce dwarf phenotypes. Recently, Hu et al. [32] demonstrated editing *aminocyclopropnae-1-carboxylase oxidase* (*MaACO1*) in banana extended shelf-life through reduced ethylene synthesis. *MaACO1* encodes for an O2-activating ascorbate-dependent non-heme iron enzyme that catalyzes the last step in ethylene biosynthesis.

**Figure 2:**
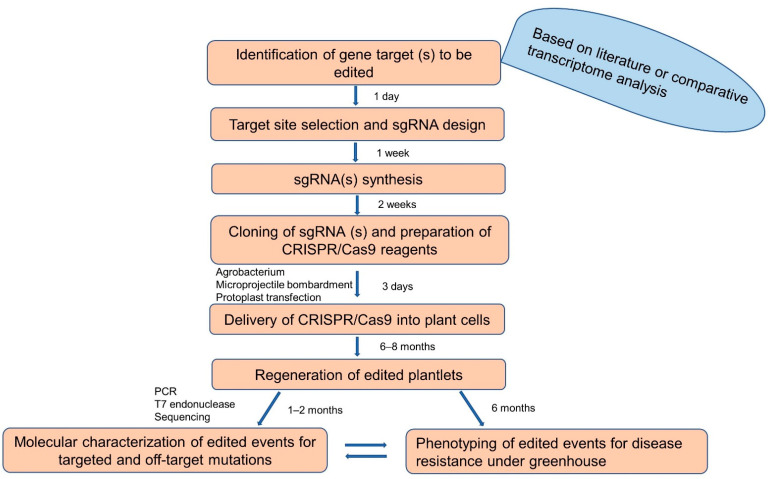
Flowchart illustrating steps and approximate time needed to develop gene-edited banana. sgRNA- synthetic guide RNA.

CRISPR/Cas9-based gene editing has been recently optimized for banana crops in several labs, facilitating functional genomics to identify defense genes responsible for disease-resistant traits. It takes about 13–15 months from target gene identification to generation and phenotyping of gene-edited banana plants with resistance to a bacterial pathogen (Figure 2).

## 7. Strategies for Developing Bacterial Wilt Resistant Banana

Gene editing offers a cost-effective mechanism for generating disease-resistant banana cultivars. The development of disease-resistant varieties has been an efficient and environmental-friendly strategy for managing plant diseases. Pathogen infection and symptoms development require the coordinated activation/or repression of genes in the plant genome. Such genes must be identified and manipulated for knockout, activation, or overexpression. Target genes identification is based on revealing and comprehending cellular pathways that contribute to disease progression, as well as identifying prospective genes or proteins that, when knocked out, activated, or overexpressed, will produce the desired effect. The target genes that are likely to be regulated upon pathogen infection can be identified in various ways. One way is through comparative transcriptome analysis (RNA-seq), studying differential gene expression among the susceptible and resistant populations upon pathogen infection. 

The disease-resistant varieties could play a significant role in controlling the BXW disease in East Africa. However, no known source of resistance against the bacterial pathogen within *Musa* germplasm has been identified, except for the wild-type diploid banana progenitor “*Musa balbisiana*” [4]. The knowledge of disease resistance mechanisms in wild-type banana against a bacterial pathogen can be used to develop resistance by editing genes related to susceptibility and/or negative regulation of plant immunity or activating the defense genes. To identify the *Musa* genes for developing the BXW-resistant varieties, we investigated the molecular mechanism of disease resistance in banana progenitor “*Musa balbisiana”* [4]. To further understand the pathogen response, the transcriptome of highly susceptible banana cultivar “Pisang Awak”, challenged with bacterial pathogen Xcm, was compared with that of disease-resistant genotype “*Musa balbisiana”* infected with Xcm. The differentially expressed genes were linked to the genetic map associated with the biotic stress and are an important part of the biotic pathway. This study identified several genes involved in the disease resistance (R) protein-mediated defense and activation of pathogen-associated molecular patterns (PAMP)-triggered basal defense in “*Musa balbisiana”* as an early response to Xcm infection [4]. We observed that most of the susceptibility genes that facilitate pathogen growth and infection were highly upregulated, and those associated with response to biotic stress were downregulated in the susceptible cultivar in comparison to the resistant cultivar [4]. These differentially expressed genes, as well as those identified in other crops (Table 1), could be knocked out, activated, or overexpressed in the susceptible cultivar to enhance resistance to BXW. 

Another way of identifying potential targets for editing or overexpression is through literature search. The molecular basis of plant immunity has been extensively studied within the last three decades, and the genes involved in resistance have been identified, cloned, characterized, and well documented in many plants [33]. Several genes have been used to develop resistance to pathogens in several crops, either by classical genetic engineering or by gene editing (Table 1). Banana can be engineered with some of those genes to develop varieties with increased resistance to BXW [34,35,36]. Under field trial in Uganda, banana overexpressing *hypersensitive response-assisting protein* (*Hrap*) or *plant ferredoxin-like protein* (*Pflp*) genes from sweet pepper (*Capsicum annuum*) demonstrated increased BXW resistance through various crop generations and agronomic performance comparable to non-transgenic control banana [34]. 

Furthermore, transgenic banana constitutively expressing *rice pattern recognition receptor* (PRR), *Xa21*, showed enhanced resistance to BXW disease [36]. The commercialization of transgenic modification is a major issue because of continuous issues regarding regulatory and public acceptance. Recent advances in gene editing have the potential to accelerate the breeding of banana by making targeted, precise changes efficiently in the plant genome to develop genotypes with increased resistance to diseases.

**Table 1:** Summary of potential target gene that could be manipulated in banana to develop resistance against bacterial pathogens.

Mode of Action	Potential Target Gene	Type of Manipulation	References
Hypersensitivity response	*Hrap*	Overexpression	[34]
	*Pflp*	Overexpression	[34]
	*Stacked Hrap and Pflp*	Overexpression	[35]
Pathogen recognition receptors induced immunity	*Xa21*	Overexpression	[36]
Susceptibility genes	*MusaDMR6*	Gene knockout	[11]
	*SlDMR6*	Gene knockout	[37]
	*OsSWEET14*	Gene knockout	[38]
	*OsSWEET11, OsSWEET13 OsSWEET14 promoter*	Gene knockout	[39]
	*DIPM1, DIPM2, DIPM4*	Gene knockout	[40]
	*CsLOB1/CsLOB1promoter*	Gene knockout	[41,42]
	*MLO*	Gene knockout	[43]
	*AtAN9*	Gene knockout	[37]
	*OsXa13*	Gene knockout	[44]
	*OsCul3a*	Gene knockout	[45]
Protein kinases as a negative regulator of plant defense	*OsMPK5*	Gene knockout	[46]
Nutrient transporter	*ENOD*	Gene knockout	[4]
E3 ubiquitin-protein ligase	*PUB*	Gene knockout	[4]
Pathogen-associated molecular patterns	*LRR*	Overexpression/CRISPR activation	[4]
Receptor kinases	*WAK2*, *WAK5*	Overexpression/CRISPR activation	[4]
Antimicrobial peptides	*Vicilin*	Overexpression/CRISPR activation	[4]
Resistance proteins	*RPM1*	Overexpression/CRISPR activation	[4]
Defense signaling	*PR1*	Overexpression/CRISPR activation	[4]
	*NPR1*	Overexpression/CRISPR activation	[47]

## 8. Knockout of Susceptibility Genes

Susceptible (*S*) genes are endogenous plant genes that aid pathogen proliferation, infection, and symptom development during colonization [3,4,11,48]. The loss of function of these genes may induce recessive resistance to plant diseases. Plants with S-gene-targeted resistance may have long-lasting immunity. S gene-based resistance is achieved by the inactivation of a host factor that is necessary for a pathogen’s survival in the host. The pathogen, therefore, must create the same or equivalent activities to overcome S gene-based resistance and infect the plant [49]. The modification or deletion of host susceptibility genes could thus be a viable technique for achieving bacterial resistance by inactivating the pathogen. Editing of S genes in crops has been reported to enhance resistance against the particular pathogen and even broad-spectrum resistance in some cases [50]. However, S genes are often pathogen-specific and, therefore, crucial to identify and target the relevant S gene(s) while attempting to acquire resistance capacity against a given disease. *Mildew Locus O* (*MLO*) was the first S-gene identified in spring barley in the 1940s and later employed in other plant species for resistance against pathogens [51,52]. It is a negative regulator of plant defense. However, loss of function of *MLO* function might lead to a trade-off between growth and yield [51]. 

S-genes have been classified into three categories based on the mode of function [53,54]. The first category includes genes required for the pathogen to recognize the host; an example is S-gene encoding a product exploited by pathogens during infection and colonization in potato [53]. In this case impairing the S-gene results in recessive resistance traits in contrast to the dominant R-gene-mediated resistance, which is based on pathogen recognition-based resistance. The second group constitutes genes supporting pathogen needs, such as *Sugar Will Eventually be Exported Transporters* (SWEET). Plant pathogens use the SWEET family genes to encode cross-membrane sugar transporters for virulence. The activation of SWEET genes leads to the transportation of more sugar outside the cell, therefore making it available to bacteria [55]. Deleting or suppressing the induction of the SWEET genes will reduce sugar trafficking outside the cell and thus prevent pathogen colonization and infection. For example, editing of TAL effector-binding sites within the promoter of SWEET14 gene in rice by TALEN led to increased resistance to *Xanthomonas oryzae* pv. *oryzae* (Xoo) due to the absence of induction of SWEET14 by the pathogens [38].

The third group comprises genes that control plant defense response. The disease susceptibility gene for citrus bacterial canker disease *Citrus sinensis Lateral Organ Boundary1* (CsLOB1) gene encodes a transcription factor regulating plant growth and development [56]. CRISPR/Cas mutation of the effector binding site within the promoter of the CsLOB1 gene in orange conferred resistance to the bacterial canker pathogen *Xanthomonas citri* spp. *citri* (Xcc) [41]). In grapefruit (*Citrus paradisi*), altering the coding area of CsLOB1 with CRISPR-Cas resulted in increased resistance to Xcc [42].

The *downy mildew resistance 6* (DMR6) gene was recently edited in banana using the CRISPR/Cas9 technique [11]. DMR6 encodes 2-oxoglutarate Fe (II)-dependent oxygenase (2OGO), which is upregulated during pathogen infection [57]. The *Musadmr6* mutants generated showed enhanced resistance to BXW disease and without any phenotypic abnormalities [11]. Additional S-genes such as MLO, *enhanced disease resistance 1* (EDR1), *ethylene-responsive factor* (ERF), SWEET genes, and CsLOB1 would be excellent candidates for editing to develop resistance to bacterial diseases in banana [4,39,58,59,60].

## 9. Activation of Defense Genes through CRISPR Activation (CRISPRa)

CRISPR/Cas9 has revolutionized several areas of plant science by aiding the development of modern tools that address some of the limitations of classical genetic engineering. The development of inducible CRISPR/Cas9 transcriptional activator methods (CRISPRa) has much promise in terms of producing plants with excellent agronomic traits. CRISPRa is a type of CRISPR tool that combines transcriptional activators with a modified version of Cas9 that lacks the endonuclease activity (dead Cas protein; dCas) to enhance gene expression. When a deactivated version of the Cas9 protein is created by mutating its nuclease domains, CRISPR/dCas9 loses the endonuclease cleavage activity but retains the capacity to bind the targeted DNA sequence [61]. Fusion of dCas9 with activation domains allows for precise and effective transcriptional activation of any gene without introducing any mutations in the endogenous gene.

The most common type of such CRISPRa activator is VP64 (and combinations) transcriptional activator domains fused to the C-terminus of *Streptococcus pyogenes* (SP)-dCas9 and has been proven to boost endogenous expression [62]. VP64 is a tetramer of VP16–a well-characterized herpes simplex virus transcription activator. It is among the first generation of CRISPRa systems and demonstrates a strong induction of activation in many experiments [63]. First-generation CRISPRa systems contain dCas9 fused with transactivator and sgRNA. Other first-generation dCas transactivators such as p65 and p300 were also developed and used for gene activation [64,65]. Different oligomers of VP16, VP48, VP160, or VP192 have been used as activators [66,67,68,69]. 

Lowder et al. [70] reported that the endogenous genes in plants could be activated using a dCas9-VP64 system comprising of the deactivated CRISPR-dCas9 fused with four tandem repeats of the transcriptional activator VP16 or VP64. They reported the successful transcriptional activation of protein in *Arabidopsis* and tobacco. However, this first generation of CRISPRa system with single domain fusions to dCas9 showed only low/moderate activation rates in plants. Hence, the second generation CRISPRa system was developed.

The second generation of the CRISPRa system is made up of three parts: dCas9, sgRNA, and effectors, which are used in multiple copies by specific domains on dCas9 or sgRNA. This type of structure is thought to increase the efficiency of the alteration, be it activation, repression, epigenetic modifications, or something else entirely [63]. These systems include: 1. the scaffold and casilio, which is based on the scaffold RNA (scRNA). The casilio relies on a combination of CRISPR-Cas9 and the Pumilio RNA-binding protein, which includes dCas9, sgRNA with several PBSs, and PUF-domains, fused with the effectors. 2. The Synergistic Activation Mediator, based on chimeric dCas9-VP64, sgRNA with synthetic aptamers for MS2 recruitment and a chimeric MS2-p65-HSF1 activation helper protein. 3. The Supernova Tagging System, whose antibodies have high affinity and specificity for short peptide sequences. Using the second-generation system, Lowder et al. [71] developed a CRISPRa system (known as CRISPR-Act2.0) by combining VP64 by dCas9 and sgRNA2.0 and targeting the genes previously activated in *Arabidopsis* by the dCas9-VP64 system. They showed that the CRISPR-Act2.0 led to stronger transcriptional activation compared to the dCas9-VP64 system. 

Similarly, Selma et al. [72] developed a CRISPRa system containing dCasEV2.1 loaded with six sgRNAs combinations, targeting the promoter of *NbDRF* and *NbAN2* genes in *Nicotiana benthamiana*. Transgenic lines expressing the second-generation CRISPRa system showed stronger gene activation than the first-generation CRISPRa [72]. Recently, Pan et al. [73] developed a highly robust CRISPRa system for multiple genes activation in rice, tomato, and Arabidopsis. The system, which they termed CRISPR–Act3.0, was developed by testing different effector recruitment strategies and various transcription activators based on deactivated *Streptococcus pyogenes* Cas9 (dSpCas9). The CRISPR–Act3.0 system activates the genes four- to six-times more when compared to the first generation and CRISPR-Act2.0 [73].

We are currently using CRISPRa to activate the expression of the endogenous banana genes such as antimicrobial Vicilin, Leucine-Rich Repeat (LRR), Wall Associated Kinase (*Wak2* and *Wak5*) and Pathogenesis-Related (*PR*), and disease resistance R (e.g., *Musa RPM1* gene) to confer resistance to BXW. The genes were identified based on the transcriptomic analysis of BXW-resistant diploid banana progenitor “*Musa balbisiana*” and BXW-susceptible cultivar “Pisang Awak” [4]. 

The CRISPRa technique requires the continuous expression of the dCas9 fusion protein, and, therefore, will always be transgenic and requires biosafety regulations. Other strategies should need to be explored to increase expression in a non-transgenic manner, such as with promoter modifications for activation of endogenous genes.

## 10. Limitations in Gene Editing of Banana and Future Prospects

To develop the gene-edited crops, the CRISPR reagents (Cas9 and sgRNAs) are delivered to the plant cells, and then the edited cells or tissues are regenerated to develop complete plantlets. Delivery of CRISPR reagents in banana cells requires an effective transformation system. In banana, *Agrobacterium*-mediated transformation has been the most efficient technique to produce transgenic and gene-edited plants. *Agrobacterium*-mediated transformation is economical, easy to use, and generates thousands of events within a year time frame. Several researchers have reported *Agrobacterium*-mediated transformation systems of various banana cultivars using embryogenic cell suspensions [74,75,76,77]. Embryogenic cells are the preferred explant for genetic transformation and gene editing in banana. 

Gene editing of farmer-preferred varieties of banana requires an efficient genotype-dependent transformation protocol. The development of edited plants using embryogenic cell suspension is found to be genotype-dependent. Although the protocol for generating embryogenic cells is labor-intensive, time-consuming, and cultivar-dependent [77], the protocol reduces the number of chimeric plants and produces a high number of transgenic events. The major limitation of using the cell suspension-based transformation system is that many cultivars, especially the East African Highland Banana are recalcitrant to embryogenic cell production. Nevertheless, the transformation efficiency for many cultivars is still low. One of the ways to overcome this challenge in banana is to enhance the transformation efficiency using morphological regulator genes such as *Baby boom* (*Bbm*), *Wuschel2* (*Wus2*), and *Shoot Meristemless* (*Stm*). This will increase banana gene editing potential in developing resistance to bacterial diseases in the farmers’ preferred cultivars. The genetic transformation efficiency has been increased using the morphological regulators in several recalcitrant crops such as cereal crops such as maize, sorghum, and wheat [78,79]. For example, the introduction of maize *Bbm* and *Wus2* in addition to a gene of interest enabled the transformation of various commercial maize lines which had previously been non-transformable [80,81]. These maize morphogenic genes also stimulated the transformation of immature embryos in sorghum, sugarcane, rice [80], and wheat [78]. In wheat, the overexpression of a chimeric protein comprising growth-regulating factor 4 (GRF4) and its co-factor, growth-interacting factor 1 (GIF1), improved regeneration efficiency of transgenic plants and extended transformation and regeneration to known recalcitrant genotypes [82]. The *STM* is required for proposer meristem formation. In *Brassica oleracea,* STM expression increased somatic embryogenesis by two-fold, whereas in *Nicotiana tabacum, the expression of* maize STM ortholog *KNOTTED1* (*KN1*) resulted in three-times shoot organogenesis [78]. These studies show that morphogenic genes are good candidates for enhancing plant transformation and should be harnessed for banana transformation.

Another limitation is that the current gene-editing tool for banana relies on *Agrobacterium*-mediated transformation using a plasmid containing the sgRNA and Cas9 gene. The most common method of delivering CRISPR reagents into plant cells is via plasmids. However, because the transgene(s) from the plasmids integrates into the plant genome, this method produces transgenic plants, which can be eliminated by backcrossing and selecting transgene(s) free events. Removal of these foreign genes (s) by crossing is not feasible in the banana for most cultivars as they reproduce asexually. Hence, the mutated plants will be regarded as GMO by regulatory authorities, which could limit their acceptability. Therefore, there is a need to produce foreign DNA-free banana that will bypass stringent regulation and be generally accepted. Different strategies could be employed to produce DNA-free gene-edited banana plants. 

One approach is to deliver preassembled Cas9 protein-sgRNA ribonucleoproteins, otherwise known as RNPs, into plant cells [40,83,84,85,86]. These RNA-guided engineered nucleases (RGENs)-RNPs direct gene editing at target sites upon transfection, and then the reagents degraded rapidly in cells, thus leaving no traces of foreign DNA elements and reducing the chances of off-target effects [83,85]. Direct delivery of RGENs-RNPs into plant cells could be achieved through various transformation methods like particle bombardment, electroporation, and protoplast transfection by polyethylene glycol (PEG), cell-penetrating peptides, or mesoporous silica nanoparticle-mediated direct protein delivery. Although protoplasts constitute a versatile platform for the generation of DNA-free edited plants, in banana, regeneration of plants from banana protoplasts remains a challenge. Panis et al. [87], Matsumoto and Oks [88], and Assani et al. [89] have reported regeneration of plants from nurse cultures of banana protoplasts. However, the method remains a challenge as it displays protoplast instability in vitro, low regeneration rate, poor reproducibility, difficult regeneration, and high levels of off-types. 

A second approach involves the transient expression of the editing machinery into the plant cell. In the absence of antibiotic selection, *Agrobacterium* infection can be used for the transient delivery of the editing machinery and generation of DNA-free plants without integrating T-DNA into the plant genome. Transient expression of CRISPR/Cas9 containing sgRNAs targeting the *PDS* gene in tobacco resulted in 8.2% non-transgenic mutants [90]. A similar approach was adopted by Veillet et al. [91]. They performed *Agrobacterium* transient infection of potato and tomato to modify the *acetolactate synthase* (ALS) gene via a cytidine-based editor (CBE). They obtained transgene-free potato and tomato plants with 10 and 12.9% mutation efficiency, respectively. Unfortunately, there were high off-target effects suggesting the need for further protocol optimization.

The major drawback in both RNPs and transient expression is that, without the visual marker, the identification of potentials edits remains a challenge as hundreds of plants must be screened. *Agrobacterium*-mediated transformation with the use of antibiotics for selection remains the most efficient method for delivering CRISPR/Cas9 components into plants cells. However, there is a need to design plasmids with mechanisms for excision and removal of T-DNA after editing. Costa et al. [92] developed two systems for T-DNA removal in the gene-edited plants. The first system uses the application of the site-specific recombinase Flippase *(Flp),* which recognizes the 34-bp long FRT sequences (Flp/FRT) system, and the second system uses Cas9 and synthetic cleavage target sites (CTS) near T-DNA borders, which are recognized by the sgRNA. Unfortunately, they observed trimming at T-DNA borders which impaired the excision mechanisms. Nonetheless, the study is a significant advancement in the production of DNA-free gene-edited plants by *Agrobacterium*-mediated transformation with antibiotics selection.

In our laboratory, we are currently optimizing the protocol to produce DNA-free banana by *Agrobacterium* transient delivery of the Cas9-sgRNA reagent targeting the *PDS* gene. We are also developing protocols for protoplast isolation, transfection with PEG, and regeneration. If successful, it will pave the way for the production of disease-resistant foreign DNA-free edited banana. In many countries such as Argentina, Australia, Brazil, Chile, Colombia, Canada, Japan, and the USA, gene editing products, particularly SDN1, with gene knockouts but with no foreign gene integration, are not regulated [21]. These countries have developed regulatory guidelines, and gene-edited crops with no foreign-gene integration are not regulated as GMO. In Africa, Nigeria and Kenya are the only countries that have developed regulatory frameworks for gene editing products. Products, particularly SDN1 type, are not regulated if no foreign gene is integrated. Other countries like Ghana, Kenya, and South Africa, are developing the regulatory framework for the application of gene editing.

## 11. Conclusions

Bacterial diseases cause major losses in banana, particularly where bacterial pathogens coexist with other pathogens and pests. BXW disease is among the most serious biotic diseases affecting banana production in East Africa, which is the largest producer and consumer of banana in the region. The disease impacts the production of all varieties of banana grown in the region and has adversely affected the food security and income of smallholder farmers, who rely on banana for a living. Currently, bacterial diseases are mainly managed by following phytosanitary practices; however, because these techniques are labor-intensive, their adoption has been inconsistent. The use of disease-resistant varieties is a productive and cost-effective strategy for managing plant diseases. Research for developing bacterial disease resistance is quite limited, particularly for moko, bugtok, and blood diseases. Some efforts are in place for the control of BXW disease.

Recent advances in CRISPR/Cas-based gene-editing techniques in banana can enhance the development of disease-resistant varieties. We are currently advancing the application of CRISPR/Cas9-mediated gene editing to control BXW disease by interrupting the function of disease-causing susceptibility (S) genes, negative regulators of plant defense, or nutrient transporters. The target genes were identified based on the literature or comparative transcriptomic analysis of BXW-resistant wild-type banana “*Musa balbisiana*” and BXW-susceptible banana cultivar at early infection with Xcm. Recently, we showed that the knocking down of the banana orthologue of the *downy mildew resistance* 6 (*MusaDMR*6) gene conferred enhanced resistance to BXW disease. 

Gene editing has the potential to revolutionize food production using the available resources. Gene-edited improved varieties of various crops can potentially be released to the farmers without going through the same lengthy regulatory process required for GM crops. Gene-edited crops with no foreign gene integration are not regulated as GMOs in several countries. The bacterial-disease-resistant banana varieties can contribute to global food security and address the challenges of feeding the growing human population. Banana genetic improvement holds excellent prospects for improving food security because it provides food to more people per unit area of production than other staple crops, especially in Africa.

## Data Availability

Not applicable.
